# Genome-wide detection of genetic structure and runs of homozygosity analysis in Anhui indigenous and Western commercial pig breeds using PorcineSNP80k data

**DOI:** 10.1186/s12864-022-08583-9

**Published:** 2022-05-17

**Authors:** Yao Jiang, Xiaojin Li, Jiali Liu, Wei Zhang, Mei Zhou, Jieru Wang, Linqing Liu, Shiguang Su, Fuping Zhao, Hongquan Chen, Chonglong Wang

**Affiliations:** 1 Key Laboratory of Pig Molecular Quantitative Genetics of Anhui Academy of Agricultural Sciences, Anhui Provincial Key Laboratory of Livestock and Poultry Product Safety Engineering, Institute of Animal Husbandry and Veterinary Medicine, Anhui Academy of Agricultural Sciences, Hefei, 230031 China; 2 Key Laboratory of Animal Genetics, Breeding and Reproduction (Poultry) of Ministry of Agriculture, Institute of Animal Science, Chinese Academy of Agricultural Sciences, Beijing, 100193 China; 3 College of Animal Science and Technology, Anhui Agricultural University, Hefei, 230036 China

**Keywords:** Anhui indigenous pig breeds, genetic structure, runs of homozygosity, Inbreeding coefficient, ROH island

## Abstract

**Background:**

Runs of homozygosity (ROH) are continuous homozygous regions typically located in the DNA sequence of diploid organisms. Identifications of ROH that lead to reduced performance can provide valuable insight into the genetic architecture of complex traits. Here, we systematically investigated the population genetic structure of five Anhui indigenous pig breeds (AHIPs), and compared them to those of five Western commercial pig breeds (WECPs). Furthermore, we examined the occurrence and distribution of ROHs in the five AHIPs and estimated the inbreeding coefficients based on the ROHs (F_ROH_) and homozygosity (F_HOM_). Finally, we identified genomic regions with high frequencies of ROHs and annotated candidate genes contained therein.

**Results:**

The WECPs and AHIPs were clearly differentiated into two separate clades consistent with their geographical origins, as revealed by the population structure and principal component analysis. We identified 13,530 ROHs across all individuals, of which 4,555 and 8,975 ROHs were unique to AHIPs and WECPs, respectively. Most ROHs identified in our study were short (< 10 Mb) or medium (10–20 Mb) in length. WECPs had significantly higher numbers of short ROHs, and AHIPs generally had longer ROHs. F_ROH_ values were significantly lower in AHIPs than in WECPs, indicating that breed improvement and conservation programmes were successful in AHIPs. On average, F_ROH_ and F_HOM_ values were highly correlated (0.952–0.991) in AHIPs and WECPs. A total of 27 regions had a high frequency of ROHs and contained 17 key candidate genes associated with economically important traits in pigs. Among these, nine candidate genes (*CCNT2*, *EGR2*, *MYL3*, *CDH13*, *PROX1*, *FLVCR1*, *SETD2*, *FGF18*, and *FGF20*) found in WECPs were related to muscular and skeletal development, whereas eight candidate genes (*CSN1S1*, *SULT1E1*, *TJP1, ZNF366*, *LIPC*, *MCEE*, *STAP1*, and *DUSP*) found in AHIPs were associated with health, reproduction, and fatness traits.

**Conclusion:**

Our findings provide a useful reference for the selection and assortative mating of pig breeds, laying the groundwork for future research on the population genetic structures of AHIPs, ultimately helping protect these local varieties.

**Supplementary Information:**

The online version contains supplementary material available at 10.1186/s12864-022-08583-9.

## Introduction

Runs of homozygosity (ROH) are defined as contiguous homozygous genotype segments present in an individual due to the parents transmitting identical haplotypes to their offspring [1]. Long ROHs are associated with more recent inbreeding within a pedigree, whereas short ROHs are associated with ancient common ancestors [2]. Bosse et al*.* [3] and Herrero et al*.* [4] used ROHs to investigate the population relationships, evolutionary history, and inbreeding effects in pigs. Several factors can influence the generation of ROHs, such as inbreeding, natural and artificial selection, genetic drift, and population bottlenecks. Of these, inbreeding is considered the most important factor [5]. Inbreeding leads to an increased risk of homozygosity for deleterious alleles throughout the genome, largely in the form of ROHs causing inbreeding depression, eventually leading to decreased fertility, viability, and phenotypic variation in the offspring [6]. Therefore, to avoid inbreeding depression in animal breeding programmes, a highly sensitive and accurate estimation of the inbreeding coefficient is of utmost importance [7].

Traditionally, the inbreeding coefficient has been estimated based on pedigree information (F_PED_), whose accurate estimation relies heavily on the accuracy, completeness, and depth of pedigree information. However, pedigree errors are common in many livestock populations [8]. Several alternative methods have been proposed to estimate the genomic inbreeding coefficient (genomic F) based on the development of genotype-based microarrays using single nucleotide polymorphisms (SNPs). These include the genomic relationship matrix (F_GRM_), homozygosity (F_HOM_), and ROH (F_ROH_). The genomic coefficients derived from animals/populations can be calculated without pedigree records or incomplete pedigree information. In addition, genomic F may provide a more accurate measure of inbreeding levels, even with missing pedigree information [9, 10]. Furthermore, compared with other genomic F indices, F_ROH_ is the most powerful and accurate method for detecting inbreeding effects and is closest to the true inbreeding coefficient [11, 12]_._ Thus, F_ROH_ has been widely used to estimate genomic inbreeding in livestock in recent years [13].

In pigs, ROH can also be used to estimate the inbreeding coefficient in the absence of pedigree records. To date, ROH has been used to estimate inbreeding in several Western commercial pig breeds (WECPs), including Landrace (LAN) [14], Large White (LWY) [11], Piétrain (PIE) [15], and Duroc (DUC) breeds [16], as well as Chinese indigenous pig breeds, such as the Laiwu [17], Songliao black [18], Jinhua [19], Diannan small-ear [20], and Liangshan [21] breeds. Genomic regions with a high frequency of ROH (ROH islands) can also be used to detect associations between genes and economically important porcine traits. Previous reports have identified many genes associated with pig reproduction, meat quality, fat deposition, and disease resistance traits in ROH islands [17, 20, 22]. The presence of ROH islands in the porcine genome suggests the occurrence of selection for economically important traits and environmental adaptation.

Although ROH has been used for breeding estimates in many Western commercial and Chinese indigenous pig breeds, it has been used less frequently in Anhui indigenous pig breeds (AHIPs), including the Wei (YZ), Wannan black (WNHZ), Huai (HZ), Wannanhua (WNHUAZ), and Six White (LB). These breeds have improved meat quality [23], disease resistance [24], and high fertility [25] compared with major commercial lean pig breeds. Nevertheless, the number of AHIPs has declined sharply in the past 20 years due to the large number of Western pig breeds that have been imported to improve leanness in pork (China National Commission of Animal Genetic Resources, 2011). The African swine fever disease outbreak also caused problems for the breeding programmes. Thus, this study had the following aims: (1) to detect the differences in genetic structure between AHIPs and WECPs, including 150 AHIPs (YZ, WNHZ, HZ, WNHUAZ, and LB) and 170 WECPs (LAN, DUR, PIE, LWY, and BER (Berkshire)) using the Illumina porcine 80 K SNP BeadChip; (2) to identify the occurrence and distribution of ROHs in WECPs and AHIPs; (3) to calculate and compare the genomic inbreeding coefficients (F_ROH_) between WECPs and AHIPs using ROHs; (4) to identify and compare potential ROH regions associated with economically important traits in AHIPs and WECPs. Our results could help preserve the genetic diversity of AHIPs, promoting sustainable breeding programmes for genetic improvement in these breeds.

## Results

### Analysis of population genetic structure of ten pig populations

**Fig. 1:**
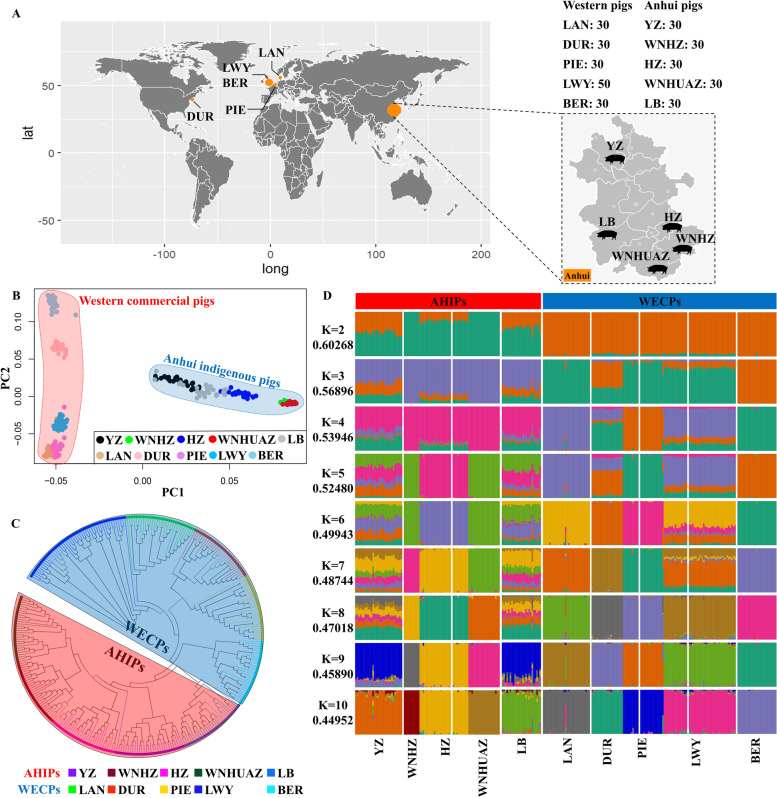
Sample information and the population genetic structure of ten pig breeds. **A** Geographical distributions and sample numbers of the ten pig breeds. **B** Principal component analysis plot for the ten pig breeds. **C** Phylogenetic tree of the ten pig breeds. **D** Admixture results (K = 2–10) for the genetic structure of ten pig breeds. The numbers on the left (under K = N) indicate cross-validation (CV) error values. Red shaded regions in (B) and (C) represent the WECPs, and blue-shaded regions represent the AHIPs. Anhui indigenous pig breeds (AHIPs): YZ, Wei pigs; WNHZ, Wannan black pigs; HZ, Huai pigs; WNHUAZ, Wannanhua pigs; LB, Six White pigs. Western commercial pig breeds (WECPs): LAN, Landrace pigs; DUR, Duroc pigs; PIE, Piétrain pigs; LWY Large White pigs; BER, Berkshire pigs

Using the genetic background information of the ten pig breeds, we examined the relatedness among populations of indigenous breeds (YZ, WNHZ, HZ, WNHUAZ, and LB) collected from Anhui Province, China. In addition, samples were collected from the five WECPs (LAN, LWY, BER, PIE, and DUR) and comparatively analysed (Fig. 1A). Principal component analysis (PCA) results and phylogenetic trees were used to visualise the genetic relationships among the ten breeds (Fig. 1B, C). The PCA results showed that the AHIPs and WECPs were clearly segregated along the PC1 axis. Furthermore, the five AHIP breeds were separated into four clusters, with WNHZ and WNHUAZ populations being classified together. Among the WECPs, the BER, DUR, and LWY pigs clustered separately, whereas the LAN and PIE pigs clustered together. The phylogenetic tree had patterns similar to those of the PCA results, showing that overall, the AHIPs and WECPs were distinguishable at the genomic level (Fig. 1C). The population genetic structure of the ten pig breeds (K = 2–10 clusters) is illustrated in Fig. 1D. Based on the cross-validation (CV = minimal) error, we identified an optimal value of K = 10 clusters, using which all ten pig breeds were clustered separately from each other. Using a K = 2, all pig breeds were collectively separated into two distinct clusters—AHIPs and WECPs. Taken together, the analysis results showed that the five AHIPs were closely related but had different genetic backgrounds, whereas the AHIPs and WECPs significantly differed.

### Distribution of runs of homozygosity

**Fig. 2:**
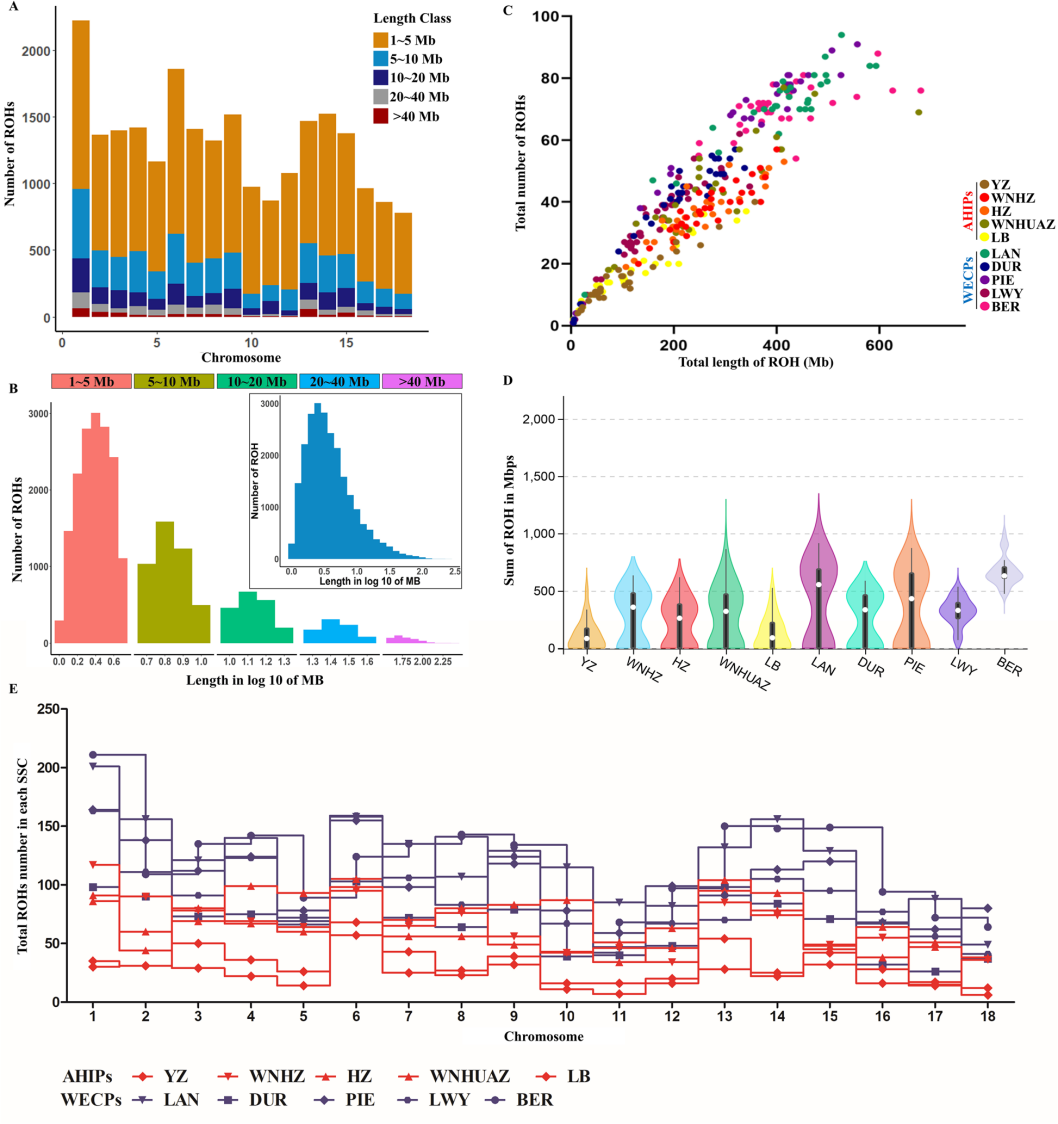
Distribution of runs of homozygosity in pig breeds. **A** Frequency distribution of the average number of ROHs in different length categories (Mb) in each pig breed. **B** Frequency distribution of the average number of ROHs in different length categories (Mb) in each chromosome. **C** Total genomic length (Mb) covered by ROHs in each individual (x-axis) and the total number of ROHs per individual (y-axis). **D** Total number of ROHs in each pig breed. **E** Total number of ROHs in each of the 18 chromosomes in each pig breed. YZ, Wei pigs; WNHZ, Wannan black pigs; HZ, Huai pigs; WNHUAZ, Wannanhua pigs; LB, Six White pigs; LAN, Landrace pigs; DUR, Duroc pigs; PIE, Piétrain pigs; LWY Large White pigs; BER, Berkshire pigs;

A descriptive summary of the ROH numbers and length categories (1–5 Mb, 5–10 Mb, 10–20 Mb, 20–40 Mb, and > 40 Mb) in each pig breed is listed in Table 1 and illustrated in Fig. 2. All the LWY individuals exhibited at least one ROH longer than 1 Mb. Among the 13,530 ROHs identified, the majority were below 10 Mb in length, accounting for approximately 97.75% of the total ROHs (1–5 Mb: 56.05%; 5–10 Mb: 31.48%; 10–20 Mb: 10.21%; 20–30 Mb: 2.17%; > 40 Mb: 0.08%) (Table 1, Fig. 1A). Moreover, the average ROH length was highest in HZ pigs (7.51 ± 0.28 Mb) and lowest in LWY pigs (4.86 ± 0.11 Mb). The average number of ROHs per pig was highest in BER pigs (70.20 ± 1.36; range, 54–88) and lowest in YZ pigs (15.00 ± 1.54; range, 4–40). The number of ROHs per chromosome tended to increase with chromosome length and was lowest on SSC11 and highest on SSC1 (Fig. 2B). Some BER, LAN, PIE, and WNHUAZ individuals had extremely long ROHs (> 500 Mb) (Fig. 2C, D); in particular, one WHHUAZ individual had an ROH covering a total length of > 600 Mb. Compared to the WECPs, the AHIPs exhibited fewer total ROHs per individual (Fig. 1A). We also examined the total ROH numbers in each chromosome for all ten pig breeds (Fig. 2E). Compared to the AHIPs, the WECPs contained more ROH fragments in all 18 chromosomes. Furthermore, the AHIPs had a lower proportion of short ROH fragments in the length categories of 1–5 Mb (29.79%), 5–10 Mb (34.94%), and 10–20 Mb (45.88%), while a higher proportion of length categories of 20–40 Mb (56.12%) and > 40 Mb (81.82%), suggesting recent inbreeding events (Table 1). Additionally, the percentage of chromosome coverage by ROH in each breed is summarised in Table S1 and illustrated in Figure S1. Among the WECPs, the highest chromosome coverage by ROH was found in PIE (SSC18: 31.3%) and the lowest in SSC13 of LWY (SSC13: 5.4%). As for AHIPs, the highest was on chromosome 17 in WNHUAZ (29.6%), while the lowest was on chromosome 1 in LB (3.5%).

**Table 1:** Summary of the number of runs of homozygosity (ROH) in different categories in each breed

Breed	N^a^	SNPs N^b^	Average Length (Mb)		Average Number		Categories (Mb)				
			**Mean ± SE**	**Range**	**Mean ± SE**	**Range**	**1–5**	**5–10**	**10–20**	**20–40**	** > 40**
**YZ**	30	50–2607	6.27 ± 0.376	1.00 – 119.46	15.00 ± 1.54	4–40	218	145	58	19	3
**WNHZ**	30	50 – 2497	7.05 ± 0.242	1.04 – 120.98	38.20 ± 1.47	20–57	512	425	164	43	2
**HZ**	30	50 – 3084	7.51 ± 0.283	1.85 – 186.91	36.33 ± 1.63	19–53	506	370	181	32	1
**WNHUAZ**	30	50 – 2135	6.30 ± 0.206	1.00 – 113.53	44.00 ± 2.96	10–77	727	391	160	40	1
**LB**	30	50 – 3553	6.56 ± 0.420	1.02–192.42	18.57 ± 1.75	2–37	296	157	71	31	2
**LAN**	30	50 – 2309	6.41 ± 0.157	1.01–104.89	71.63 ± 2.82	10–94	1177	691	218	33	NA
**DUR**	30	50 – 1993	5.27 ± 0.135	1.01 – 75.95	40.03 ± 2.70	1–57	743	349	96	12	1
**PIE**	30	50 – 2107	5.62 ± >0.139	1.01–102.87	63.27 ± 4.50	1–91	1153	591	133	21	NA
**LWY**	50	50 – 3598	4.86 ± 0.110	1.00–134.02	33.02 ± 2.05	1–62	1085	439	113	14	NA
**BER**	30	50 – 3821	6.41 ± 0.194	1.44–225.01	70.20 ± 1.36	54–88	1167	701	188	49	1

Inbreeding coefficient of ROH (F_ROH_) and homozygotes (F_HOM_)

^a^Number of samples, N

^b^Number of SNPs, SNPs N

**Fig. 3:**
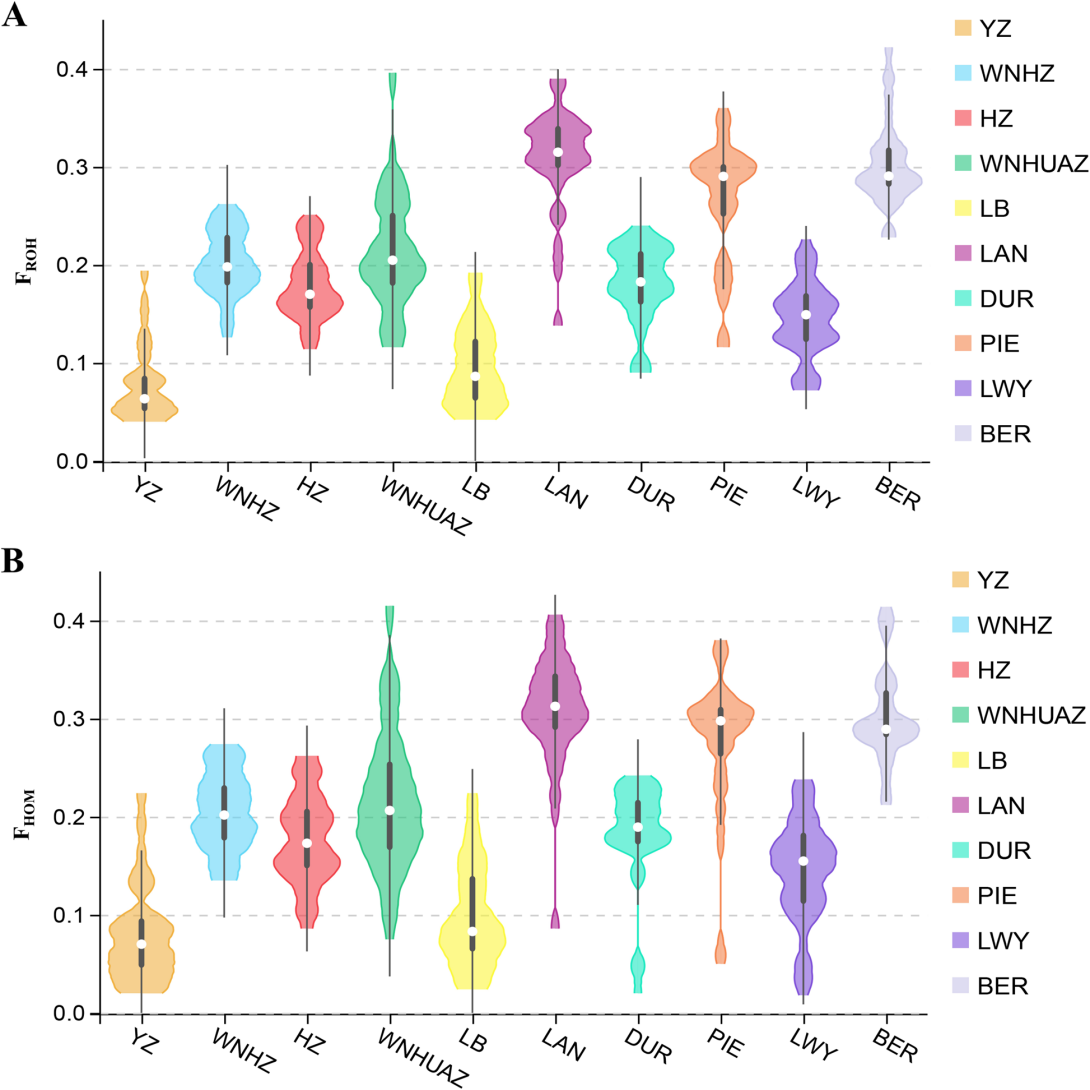
Inbreeding coefficient of ROH (F_ROH_) and homozygotes (F_HOM_) in ten pig breeds. **A** Distribution of F_ROH_ in the ten pig breeds (Mb). **B** Distribution of F_HOM_ in the ten pig breeds. YZ, Wei pigs; WNHZ, Wannan black pigs; HZ, Huai pigs; WNHUAZ, Wannanhua pigs; LB, Six White pigs; LAN, Landrace pigs; DUR, Duroc pigs; PIE, Piétrain pigs; LWY Large White pigs; BER, Berkshire pigs

The descriptive statistics for ROH-based (F_ROH_) and homozygous-based (F_HOM_) inbreeding coefficients in different length categories are listed in Table 2 and illustrated in Fig. 3. The inbreeding coefficient of F_HOM_ varied from 0.0971 ± 0.0531 (LB) to 0.3079 ± 0.0492 (LAN), and the values of F_ROH(ALL)_ varied from 0.064 ± 0.007 (YZ) to 0.289 ± 0.008 (LAN). We also found a high correlation between F_HOM_ and F_ROH_ in all ten breeds (range, 0.947–0.991), and the average correlation between F_ROH_ and F_HOM_ in the ten breeds was 0.967. The genomic inbreeding coefficients (F_ROH_ and F_HOM_) were highest in the LAN, BER, and PIE breeds of WECPs, and lowest in the HZ and YZ breeds of AHIPs (Fig. 3A, B). Similar conclusions drawn from F_ROH_ and F_HOM_ estimates indicated a considerable difference in genomic inbreeding coefficients among the different pig breeds. Of note, WECPs had significantly higher genomic inbreeding coefficients than the AHIPs. These results showed that the F_ROH_ values differed significantly between the WECP and AHIP pig breeds, indicating differences in directional selection and breeding goals.

**Table 2:** Descriptive statistics for runs of homozygosity (ROH) and inbreeding coefficients (F) in each breed

Breed	F_ROH_ (Mb, Mean ± SE)						*F*_HOM_	r (F_ROH,_ F_HOM_)
	**1–5**	**5–10**	**10–20**	**20–40**	** > 40**	**All**		
YZ	0.046 ± 0.007	0.035 ± 0.006	0.030 ± 0.006	0.022 ± 0.004	0.021 ± 0.001	0.064 ± 0.007	0.0800 ± 0.0503	0.991
WNHZ	0.120 ± 0.006	0.094 ± 0.005	0.050 ± 0.004	0.022 ± 0.002	0.020	0.180 ± 0.003	0.2046 ± 0.0399	0.952
HZ	0.111 ± 0.006	0.086 ± 0.006	0.047 ± 0.005	0.021 ± 0.003	0.024	0.151 ± 0.002	0.1783 ± 0.0468	0.971
WNHUAZ	0.120 ± 0.011	0.086 ± 0.010	0.046 ± 0.007	0.024 ± 0.006	0.021	0.190 ± 0.010	0.2163 ± 0.0733	0.969
LB	0.057 ± 0.007	0.048 ± 0.007	0.033 ± 0.005	0.025 ± 0.003	0.021 ± 0.002	0.079 ± 0.007	0.0971 ± 0.0531	0.990
LAN	0.181 ± 0.010	0.130 ± 0.008	0.059 ± 0.005	0.017 ± 0.002	NA	0.289 ± 0.008	0.3079 ± 0.0610	0.967
DUR	0.092 ± 0.007	0.064 ± 0.005	0.027 ± 0.003	0.016 ± 0.002	0.018	0.165 ± 0.005	0.1809 ± 0.0549	0.947
PIE	0.152 ± 0.012	0.103 ± 0.009	0.043 ± 0.004	0.016 ± 0.001	NA	0.241 ± 0.009	0.2654 ± 0.0889	0.953
LWY	0.073 ± 0.005	0.046 ± 0.004	0.021 ± 0.003	0.015 ± 0.005	NA	0.123 ± 0.004	0.1443 ± 0.0532	0.968
BER	0.183 ± 0.008	0.128 ± 0.009	0.055 ± 0.008	0.026 ± 0.004	0.022	0.266 ± 0.001	0.3028 ± 0.0492	0.969

*r*(*F*_*ROH*_, *F*_*HOM*_), correlation between *F*_*ROH*_ and *F*_*HOM*_; NA, no ROH was detected

*Genomic regions with a high frequency of ROHs*

**Fig. 4:**
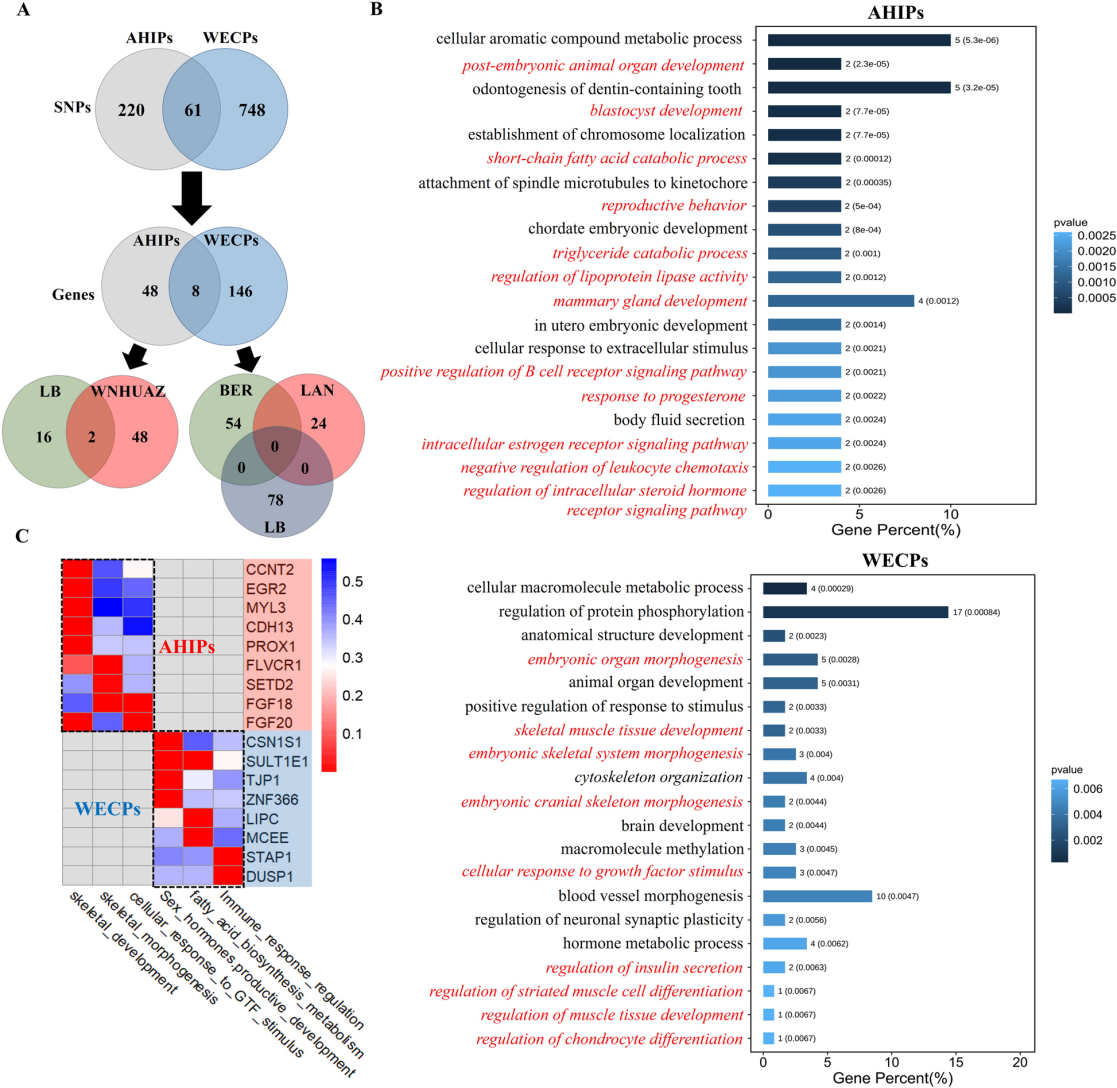
Comparisons of the annotated genes in ROH hotspots in AHIP and WECP pigs. **A** Venn diagram of SNPs and related genes in ROH hotspots in AHIPs and WECPs. **B** Gene ontology (GO) analysis of the annotated genes in ROH hotspots in AHIPs and WECPs. Red italics represent GO terms associated with economically important traits in pigs. **C** The summary of GO analysis result (Fig. **B**) in 17 key candidate genes between AHIPs (eight) and WECPs (nine). The horizontal axis represents the gene name, and the vertical axis represents the GO term (selected from Fig. **B**) related to key candidate genes. The legend represents the *P*-value of the GO term. AHIPs, Anhui indigenous pig breeds; WECPs, Western commercial pig breeds

We identified the genomic regions most commonly associated with ROHs in the ten pig breeds and plotted the percentages of SNPs in ROHs against the positions of the SNPs along the chromosomes (Supplementary Fig. 2). No ROH islands were found in the LB and YZ breeds. High percentages of SNPs in ROHs were found in the BER (SSC1, SSC3, SSC6, SSC9, SSC12, SSC15), PIE (SSC4), and LAN (SSC7, SSC10, SSC14, SSC15) breeds. The longest ROH island (6.27 Mb) was found in the WNHUAZ breed on SSC1, whereas the shortest (0.05 Mb) was found in the HZ breed on SSC8. The SNPs in ROH islands were compared between WECPs and AHIPs, and 220 and 748 unique SNPs were found in AHIPs and WECPs, respectively (Fig. 4A). A total of 27 genomic regions had a high frequency of ROHs (Table S2) and were found to contain 202 genes. Among these, 48 candidate genes were found only in AHIPs, and 146 were found only in WECPs (Fig. 4A). In addition, we aligned all of these ROH islands to the pig quantitative trait loci (QTL) database, revealing that meat-, carcass-, and production-related QTLs were enriched in 20 WECP genomic regions, while reproduction-, fatness-, and health-related QTLs were enriched in 7 AHIP genomic regions (Table S3).

### GO enrichment analysis of candidate genes in WECPs and AHIPs

Gene Ontology (GO) enrichment analysis was performed separately for WECPs and AHIPs (Fig. 4B). Genes enriched in AHIPs were mainly involved in *blastocyst development*, *response to progesterone*/*oestrogen*, *positive regulation of B cell receptor signalling pathwa*y, and *triglyceride catabolic process*, whereas those in WECPs were involved in *skeletal muscle tissue development*, *embryonic skeletal system morphogenesis*, and *cellular response to growth factor stimulus*. Furthermore, nine candidate genes (*CCNT2*, *EGR2*, *MYL3*, *CDH13*, *PROX1*, *FLVCR1*, *SETD2*, *FGF18*, and *FGF20*) in WECPs were found to be closely associated (*P*_*adj*_ < 0.05) with *skeletal muscle tissue development*, *embryonic skeletal morphogenesis*, and *cellular response to growth factor stimulus*. Eight candidate genes (*CSN1S1*, *SULT1E1*, *TJP1, ZNF366*, *LIPC*, *MCEE*, *STAP1*, and *DUSP*) related to *sex hormones and reproductive development*, *fatty acid biosynthesis metabolism*, and *immune response regulation* were selected for subsequent analyses (Fig. 4C). Similarly, QTL enrichment results also revealed that ROH islands in two AHIP breeds (WHHUAZ and HZ) were associated with QTLs of economically important traits such as health, reproduction, and fatness, whereas those in the WECP breeds were related to meat- and production-related traits (Table 3).

**Table 3:** Candidate genes located in genomic regions with a high frequency of ROH associated with pig economic traits

Breed	CHR	Start (Mb)	End (Mb)	No SNPs	No genes	Candidate genes	Gene function	Traits related to QTL	
BER	6	5.52	6.51	52	5	CDH13	Meat	Lean meat percentage (7632)	
	9	129.04	130.63	81	9	PROX1	Meat	Carcass weight (12,786)	
						FLVCR1	Meat/ Production	Average daily gain (2896)	
	14	65.87	68.09	48	7	EGR2	Meat	NA	
	15	15.76	17.03	40	11	CCNT2	Meat	Meat colour score (3009)	
	17	4.39	5.63	54	10	FGF20	Meat/Production	Average daily gain (28,911)	
PIE	13	29.59	29.89	10	9	MYL3	Meat	Loin muscle area (5499)	
						SETD2	Production	Body weight (21,843)	
	16	49.85	52.75	60	17	FGF18	Production	Average daily gain (28,900)	
WNHUAZ	1	111.18	117.45	86	6	LIPC	Fatness	Palmitoleic acid content (168,357)	
	1	142.77	145.30	41	14	MCEE	Fatness	Intramuscular fat content (17,747)	
						TJP1	Reproduction	Gestation length (10,617)	
	8	64.83	68.48	52	6	STAP1	Health	Palmitoleic acid content (168,374)	
	16	48.62	51.50	47	15	ZNF366	Reproduction	Teat number (8812)	
						DUSP1	Health	NA	
HZ	8	66.23	67.50	25	16	SULT1E1	Reproduction/ Fatness	Teat number (124,206)	
						CSN1S1	Reproduction	Corpus luteum number (492)	

The distance between genes and ROH regions was calculated as follows: The starting coordinate of the gene minus the starting coordinate of the ROH region; all candidate genes are located in the ROH region; the number within brackets in the last column represents the QTL-ID

## Discussion

The Anhui Province is one of the top ten provinces that traditionally produce pigs in China, and it has abundant genetic resources of indigenous pig breeds (YZ, WNHZ, HZ, WNHUAZ, and LB). Due to long-term natural adaptation and artificial selection, the AHIPs have gradually evolved high fertility, high fat content, excellent meat quality [23], disease resistance [24], good maternal stability [25], and crude feed tolerance [22]. In this study, WECPs and AHIPs significantly differed in terms of genetic backgrounds, consistent with previous studies showing that pigs were domesticated in at least two separate domestication centres, Europe and Asia [26–28]. Noteworthily, artificial selection has also played a vital role in AHIPs, especially for LB/YZ breeds, as genomic information from Western breeds flowed into LB/YZ breeds. This could be because, in the past 20 years, WECPs were selected and admixed with AHIPs to increase the allelic richness and improve the breeding stock of AHIPs (China National Commission of Animal Genetic Resources, 2011). Besides, the WNHUAZ and WNHZ breeds exhibited a close genetic relationship, suggesting that both breeds may have descended from the same ancestor [29], and gradually formed two different breeds due to geographical isolation and the long-term domestication process [30]. Furthermore, the similar number of ROHs and F_ROH_ values in WNHUAZ and WNHZ also supported the notion that these breeds originated from a common population. However, due to the small sample size and marker density, the results of population genetic structure in the WECP and AHIP breeds are insufficient and need further investigation.

The abundance, length, and genomic distribution of ROHs provide valuable information about the demographic history of livestock species [3]. In this study, the occurrence and distribution of ROHs were compared between five AHIPs and five WECPs. Most of the ROHs identified in our study belonged to the short (< 10 Mb) and medium (10–20 Mb) length categories, consistent with those reported in chickens [31], sheep [32], pigs [18], and cattle [33]. The short ROHs indicate ancient inbreeding, whereas long ROHs suggest recent inbreeding [34]. Compared with WECPs, AHIPs had more ROHs in 20–40 Mb and > 40 Mb categories, fewer ROHs in 1–5 Mb and 5–10 Mb. These results are consistent with those of previous studies [18, 35]. The different distribution patterns of ROH numbers and lengths between the WECPs and AHIPs may be due to the selection of different traits in these breeds; WECP management primarily focuses on the production traits of pigs [36], whereas AHIPs are selected for meat quality and disease resistance [35].

With the development of high-throughput genotyping technologies, genetic markers can provide a more accurate estimate of population relationships in pigs than pedigree data, which may have missing or incorrect parent information [13, 22]. In recent years, ROHs have been widely used to predict inbreeding levels in livestock [13]. F_ROH_ estimates are more accurate for estimating autozygosity and detecting inbreeding effects than pedigree data [11], providing useful information about interindividual genetic relatedness. In this study, we used two indices, F_ROH_ and F_HOM_, to estimate inbreeding coefficients in AHIPs and WECPs. Previous studies have reported that F_ROH_ generally highly correlates with F_HOM_ (*r*_FROH,FHOM_ = 0.78–0.85) consistent with our results (*r*_FROH,FHOM_ = 0.952–0.991) and previous studies [18, 37]. Moreover, we found that F_HOM_ values were higher than F_ROH_ values in all ten pig breeds because the F_HOM_ estimate cannot distinguish identity by descent alleles from identity by state alleles, inevitably overestimating inbreeding levels [38]. Although using F_HOM_ to estimate the inbreeding coefficient is not sufficiently accurate, F_ROH_ can alleviate the issues mentioned above. Thus, theoretically, F_ROH_ may be a more effective and accurate alternative for quantifying relatedness and inbreeding levels [39]. Further, the F_ROH_ of AHIPs is generally expected to be lower than that of WECPs. The contradictory results of our study may be explained by the small effective population size and random sampling errors in WECPs, resulting in higher inbreeding estimates for WECPs in recent generations [40].

We found that the ROH islands harboured several candidate genes controlling economically important traits in pigs. In particular, we identified 27 genomic regions with a high frequency of ROHs, harbouring 17 key candidate genes in WECPs and AHIPs. Furthermore, we identified eight candidate genes in the AHIPs, of which three (*SULT1E1*, *LIPC*, and *MCEE*) were involved in fat deposition, three (*CSN1S1*, *TJP1*, and *ZNF366*) were involved in reproduction, and two (*STAP1* and *DUSP1*) were immune system-related. *LIPC* encodes hepatic lipase and affects the metabolism, composition, and expression of several lipoproteins [41, 42]. *SULT1E1*, a negative regulator of adipogenesis [43], serves a strong regulatory function in lipid metabolism via the PPARγ pathway [44]. *SULT1E1* is also reportedly linked to foetal development [45], and ablation of the murine *SULT1E1* gene causes placental thrombosis and spontaneous foetal loss [46]. *ZNF366* plays an important role in regulating the expression of target genes in response to oestrogen [47, 48]. *TJP1* has been related to testis weight, spermatogenesis, and the development of ovarian and cystic follicles [49, 50]. *CSN1S1* is an effective molecular marker for litter size in goat breeding [51]. *STAP1* [52] and *DUSP1* [53, 54] are significantly associated with anti-inflammatory responses and immune infiltration in human autoimmune diseases. We also identified nine candidate genes in the WECPs, of which six (*CDH13*, *PROX1*, *EGR2*, *CCNT2*, *SETD2*, and *MYL3*) were related to muscular development, and three (*FLVCR1*, *FGF18*, and *FGF20*) were involved in skeletal morphogenesis. Among these candidate genes, miR-15a [55] and miR-155-5p [56] inhibit skeletal muscle development and differentiation by targeting *CCNT2*. High expression levels of *CDH13* promote muscle-type identity, as *CDH13* plays an active role in myogenesis [57, 58]. In pigs, *MYL3* [59, 60] and *EGR2* [61, 62] are associated with muscle formation and development. *PROX1* is involved in muscle fibre conversion, and is a promising candidate gene affecting pork quality traits [59, 63]. *FGF18* [64, 65] and *FGF20* [66, 67] are reported to play important roles in embryonic development, bone growth, and bone‐related diseases. Moreover, *FLVCR1* deficiency results in Diamond–Blackfan anaemia, often associated with skeletal malformations [68]. Based on the Pig QTL database, reproduction, fatness, and health traits overlapped in the ROH islands of AHIPs, while meat- and production-related traits were observed within ROH islands of WECPs. Overall, we found that the AHIP breeds were more adapted to fat deposition, disease resistance, and high fertility, whereas WECP pigs showed selection for production traits, such as muscular and skeletal development. Taken together, our results indicate that the WECP and AHIP breeds show adaptive differences in economically important traits.

## Conclusions

In this study, we characterised the population genetic structure of WECPs and AHIPs and found that they had considerably different genetic backgrounds. Furthermore, the occurrence and distribution of ROHs were compared across five AHIPs and five WECPs. Results revealed how diversity has evolved in the AHIP populations. F_ROH_ and F_HOM_ values were significantly lower in AHIPs than in WECPs, indicating that the breeding and conservation programmes were successful in AHIPs. Several genes with a high frequency of ROHs were identified. Among these, candidate genes in AHIPs were associated with fat deposition, disease resistance, and high fertility, whereas those in WECPs were related to muscular and skeletal development. Overall, our findings provide a helpful reference for selection and assortative mating programmes in pigs. Moreover, these results reveal a novel research direction regarding the population genetic structure of AHIP breeds, which might effectively help protect these valuable local varieties.

## Methods

### SNP genotyping and quality control

A total of 320 pigs were used in this study: 170 WECPs (Duroc, Landrace, Yorkshire, Berkshire, and Piétrain pig breeds) and 150 AHIPs from the Anhui Province, China. Detailed information on the sampled pig breeds in this study, including the regions of recollection, breed names and abbreviations, and sample size, are presented in Table S4. Genomic DNA was extracted from ear tissue and genotyped with the Illumina porcine 80 K SNP BeadChip (Illumina, San Diego, CA, USA). Only autosomal SNPs were used for further analyses. The PLINK software (v1.90) [69] was used for quality control of the data, and the following standards were set: (1) SNPs with a call rate of < 0.95 and unknown positions were removed (*–geno 0.05*); (2) SNPs with a minor allele frequency of < 0.05 were removed (*–maf 0.05*); (3) data from individuals with a call rate of < 0.90 were discarded (*–mind 0.1*); (4) Hardy–Weinberg Equilibrium (HWE) *P*-value < 1 × 10^–6^ (*–hwe 0.000001*). The SNP genome coordinates were obtained from the Sus scrofa 11.1 porcine genome reference assembly. After genotype quality control, 1158 markers were excluded based on the HWE test (p ≤ 1 × 10^–6^), 7231 SNPs failed the missingness test (GENO > 0.1), 9788 SNPs failed the frequency test (MAF < 0.05), yielding 320 individuals and 54,075 SNP for further analysis.

### Population structure

The geographical distributions of five WECPs and five AHIPs were estimated using the *ggmap* package [70] in R statistical software. To illustrate the relationships among the ten pig breeds, PCA was performed using the GCTA software (–autosome –autosome-num 18 –make-grm –pca 3) [71]. A scatterplot was generated to visualise the first and second principal components based on a variance-standardised relationship matrix created using the PCA results. The ADMIXTURE software [72] was used to infer the most probable number of ancestral populations (K = 2–10) based on the SNP genotype data. A five-fold cross-validation (–cv) error for each K was used to select the optimal K. A phylogenetic tree was created for the ten pig breeds using the online tool, the Interactive Tree Of Life (iTOL, http://itol2.embl.de/personal_page.cgi) [73].

### Genomic inbreeding coefficients

ROHs were identified for each individual using the PLINK software (v1.90), which uses a sliding window technique to scan each individual’s genotype at each marker position to detect homozygous segments [39]. We defined ROHs according to the following criteria: (1) the minimum ROH length was set to 1 Mb (*–homozyg-kb 1000*); (2) a minimum of 50 consecutive SNPs were included in an ROH (*–homozyg-snp 50*), which was calculated using the equation proposed by Lencz et al*.* [74]:

$$l=\frac{{log}_{e}\frac{\alpha }{{n}_{s}\times {n}_{i}}}{{log}_{e}\left(1-het\right)}$$

where α is the percentage of false-positive ROHs (set to 0.05 in the present study), *n*_*s*_ is the number of SNPs per individual, *n*_*i*_ is the number of individuals, and *het* is the heterozygosity across all SNPs. After calculation, the minimum number of SNPs constituting an ROH was set to 50; (3) the maximum gap between consecutive SNPs was set to 1 Mb (*–homozyg-gap 1000*); (4) the minimum SNP density was set to 1 SNP every 100 kb (*–homozyg-density 100*); (5) the minimum number of SNPs in a sliding window was set to 50 (*–homozyg-snp 50*); (6) one heterozygous genotype and no more than five missing SNPs were allowed per window (*–homozyg-window-het 1*; *–homozyg-window-missing 5*); (7) the window threshold was set to 0.01 (*–homozyg-window-threshold 0.01*). In this study, we classified ROHs into five different categories according to their physical length: 1 to < 5 Mb, 5 to < 10 Mb, 10 to < 20 Mb, 20 to < 40 Mb and > 40 Mb. For each length category, we computed the frequency of ROH numbers and the average length of an ROH in every breed.

### Inbreeding coefficient of ROH

To verify the accuracy of F_ROH_, we evaluated the genomic coefficients for the ten pig breeds using two methods: (1) PLINK v1.90 software was used to measure the inbreeding coefficient based on the difference between the observed and expected numbers of homozygous genotypes (*F*_HOM_) [74]. The inbreeding coefficient for an individual (*F*_HOM_) was calculated as follows:

$${F}_{HOM}=\frac{\left(O-E\right)}{\left(L-E\right)}$$

where *L* is the number of genotyped autosomal SNPs, *E* is the number of homozygotes expected by chance, and *O* is the number of observed homozygotes. (2) Genomic inbreeding coefficients were also estimated based on ROH (*F*_ROH_). The *F*_ROH_ was calculated as follows:

$${F}_{HOM}=\frac{{\sum }_{i}{L}_{{ROH}_{i}}}{{L}_{auto}}$$

where *L*_ROHi_ is the total length of ROH_i_ on autosomes, and *L*_auto_ is the autosomal genome length covered by the SNP chip. Furthermore, the correlation between F_ROH_ and F_HOM_ for each breed was calculated using Pearson’s correlation.

### Detection of common ROHs and gene annotation

To identify genomic regions with a high frequency of ROHs, we calculated the percentage of occurrences of SNPs in ROHs by counting the number of times an SNP was detected in those ROHs across individuals. In this study, the threshold used to define an ROH hotspot in the genome was 40%, in agreement with a previous report by Rui et al*.* [18]. Adjacent SNPs over this threshold were merged into genomic regions called ROH islands [75, 76]. We used the porcine reference genome annotation file from the NCBI database (http://asia.ensembl.org/Sus_scrofa/Info/Index) to annotate the genes in the ROH islands. In addition, pig QTLdb (https://www.animalgenome.org/cgi-bin/QTLdb/SS/index) was used to annotate the genes in the ROH islands. GO enrichment analysis of genes in the ROH islands was performed using g:Profiler (https://biit.cs.ut.ee/gprofiler/gost), and terms with a *P*-value greater than 0.05 were filtered. The biological function of each annotated gene within the ROH islands was determined through an extensive literature search.

## Supplementary Information

**Additional file 1: SupplementaryFigure 1. **The percentage of chromosome coverage (%) by ROHs in each breed. YZ, Wei pigs; WNHZ, Wannan black pigs; HZ, Huai pigs; WNHUAZ, Wannanhua pigs; LB, Six White pigs; LAN, Landrace pigs; DUR, Duroc pigs; PIE, Piétrain pigs; LWY Large White pigs; BER, Berkshire pigs.

**Additional file 2: SupplementaryFigure 2. **Manhattan plot of the occurrence (%); of SNPs in ROHs in ten pig breeds.The x-axis represents the SNP genomic coordinate in each chromosome, and the y-axis shows the proportion of overlapping ROHs shared among individuals, based upon the number in population. Colourful data points indicate SNPs, and the dashed line represents the 40% threshold. YZ, Wei pigs; WNHZ, Wannan black pigs; HZ, Huai pigs; WNHUAZ, Wannanhua pigs; LB, Six White pigs; LAN, Landrace pigs; DUR, Duroc pigs; PIE, Piétrain pigs; LWY Large White pigs; BER, Berkshire pigs.

**Additional file 3: Supplemental Table 1.** The percentage of chromosome coverage (%) by ROHs in each breed. **Supplemental Table 2.** Candidate genes located in genomic regions with a high frequency of ROHs. **Supplemental Table 3.** Pig QTLs located in genomic regions with a high frequency of ROHs. **Supplemental Table 4.** The summary of Sample information for each breed.

## Data Availability

The datasets used and/or analysed during the current study are available from the corresponding author on reasonable request.

## Abbreviations

AHIPs: Anhui Indigenous Pig Breeds
WECPs: Western Commercial Pig Breeds
ROH: Runs of Homozygosity
F: Inbreeding Coefficient
SNP: Single Nucleotide Polymorphism
YZ: Wei
WNHZ: Wannan Black
HZ: Huai
WNHUAZ: Wannanhua
LB: Six White
LAN: Landrace
DUR: Duroc
PIE: Piétrain
LWY: Large White
BER: Berkshire
PCA: Principal Component Analysis
CV: Cross-validation
SSC: Sus Scrofa Chromosome
CCNT2: Cyclin T2
EGR2: Early Growth Response 2
MYL3: Myosin Light Chain 3
CDH13: Cadherin 13
PROX1: Prospero Homeobox 1
FLVCR1: FLVCR heme transporter 1
SETD2: SET domain containing 2, histone lysine methyltransferase
FGF18: Fibroblast Growth Factor 18
FGF20: Fibroblast Growth Factor 20
CSN1S1: Casein Alpha S1
SULT1E1: Sulfotransferase Family 1E, Oestrogen-preferring, Member 1
TJP1: Tight Junction Protein 1
ZNF366: Zinc Finger Protein 366
LIPC: Lipase C Hepatic Type
MCEE: Methylmalonyl-CoA Epimerase
STAP1: Signal Transducing Adaptor Family Member 1
DUSP1: Dual Specificity Phosphatase 1
