# Supplementary figures and images for: Genome-wide detection of genetic structure and runs of homozygosity analysis in Anhui indigenous and Western commercial pig breeds using PorcineSNP80k data
Source: BMC Genomics. 2022 May 17;23:373. doi: 10.1186/s12864-022-08583-9 (PMC9115978; doi:10.1186/s12864-022-08583-9)

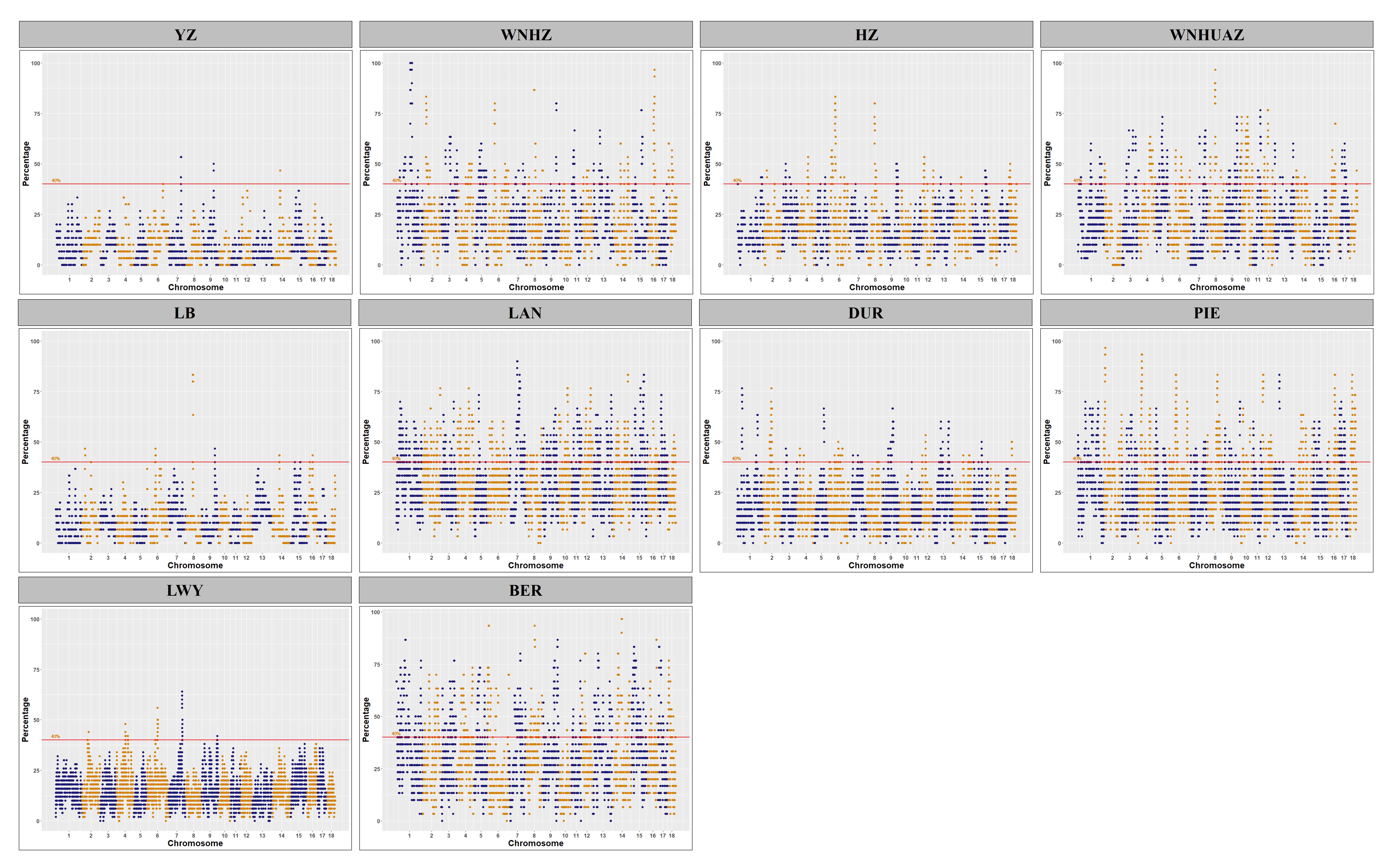

Supplement: Supplementary file 2 — Additional file 2: SupplementaryFigure 2. Manhattan plot of the occurrence (%); of SNPs in ROHs in ten pig breeds.The x-axis represents the SNP genomic coordinate in each chromosome, and the y-axis shows the proportion of overlapping ROHs shared among individuals, based upon the number in population. Colourful data points indicate SNPs, and the dashed line represents the 40% threshold. YZ, Wei pigs; WNHZ, Wannan black pigs; HZ, Huai pigs; WNHUAZ, Wannanhua pigs; LB, Six White pigs; LAN, Landrace pigs; DUR, Duroc pigs; PIE, Piétrain pigs; LWY Large White pigs; BER, Berkshire pigs. [file 12864_2022_8583_MOESM2_ESM.jpg]
