# Supplementary material for: Genome-wide detection of genetic structure and runs of homozygosity analysis in Anhui indigenous and Western commercial pig breeds using PorcineSNP80k data
Source: BMC Genomics. 2022 May 17;23:373. doi: 10.1186/s12864-022-08583-9 (PMC9115978; doi:10.1186/s12864-022-08583-9)
Supplement: Supplementary file 3 — Additional file 3: Supplemental Table 1. The percentage of chromosome coverage (%) by ROHs in each breed. Supplemental Table 2. Candidate genes located in genomic regions with a high frequency of ROHs. Supplemental Table 3. Pig QTLs located in genomic regions with a high frequency of ROHs. Supplemental Table 4. The summary of Sample information for each breed. [file 12864_2022_8583_MOESM3_ESM.docx]

**Supplemental Table 1 The percentage of chromosome coverage (%) by ROHs in each breed**

| **Breed** | **1** | **2** | **3** | **4** | **5** | **6** | **7** | **8** | **9** | **10** | **11** | **12** | **13** | **14** | **15** | **16** | **17** | **18** |
| --- | --- | --- | --- | --- | --- | --- | --- | --- | --- | --- | --- | --- | --- | --- | --- | --- | --- | --- |
| **YZ** | 5.0 | 15.1 | 14.1 | 9.6 | 6.1 | 11.4 | **4.7** | 7.5 | 10.4 | 14.1 | 8.0 | 5.7 | 5.1 | 7.3 | 10.8 | 6.5 | 10.4 | **17.2** |
| **WNHZ** | **11.5** | 14.2 | 25.8 | 13.9 | 18.0 | 12.5 | 13.8 | 12.6 | 12.8 | 14.1 | 23.4 | 22.0 | 14.4 | 13.3 | 12.7 | 17.8 | 15.0 | **25.9** |
| **HZ** | **8.7** | 15.6 | 17.9 | 16.1 | 13.1 | 14.2 | 13.8 | 13.0 | 11.6 | 17.6 | 12.0 | 13.3 | 9.7 | 15.0 | 13.7 | 12.1 | 18.6 | **24.5** |
| **WNHUAZ** | 7.8 | **5.6** | 12.7 | 22.6 | 18.1 | 15.5 | 10.6 | 12.4 | 14.1 | 27.7 | 13.5 | 16.4 | 10.5 | 16.0 | 11.2 | 21.2 | **29.6** | 23.7 |
| **LB** | 3.5 | 10.7 | 10.5 | 8.5 | **5.6** | 10.5 | 12.5 | 10.0 | 7.9 | **23.3** | 10.4 | 11.0 | 11.0 | 13.1 | 7.6 | 18.0 | 23.0 | 6.0 |
| **LAN** | 15.2 | 19.9 | 16.7 | 21.1 | 12.6 | 17.4 | 24.8 | 14.9 | 21.3 | 24.6 | 22.2 | 25.4 | **12.4** | 22.3 | 17.3 | 19.8 | **27.8** | 25.7 |
| **DUR** | **7.2** | 13.4 | 9.5 | 10.9 | 12.2 | 9.4 | 14.9 | 7.8 | 14.0 | 10.5 | 15.8 | 13.1 | 10.7 | 11.9 | 8.1 | 9.9 | 9.9 | **16.5** |
| **PIE** | 16.1 | 19.5 | 14.8 | 20.9 | 12.5 | 16.3 | 15.6 | 19.6 | 15.7 | 16.6 | 14.6 | 26.1 | **9.5** | 18.8 | 14.4 | 15.7 | 20.8 | **31.3** |
| **LWY** | 7.0 | 10.2 | 7.9 | 11.9 | 7.3 | 10.1 | 9.6 | 7.5 | 10.4 | 10.6 | 11.2 | 11.5 | **5.4** | 10.7 | 9.5 | 11.7 | **15.9** | 12.8 |
| **BER** | 17.4 | 17.6 | 18.5 | 23.1 | 18.1 | **10.5** | 20.6 | 20.1 | 21.1 | 11.1 | 16.1 | **26.9** | 13.1 | 22.9 | 25.3 | 19.5 | 21.9 | 20.9 |

Bold represents the maximum and minimum in each breed; All numbers in the table are percentages values.

**Supplemental Table 2 Candidate genes located in genomic regions with a high frequency of ROHs**

| **Population** | **CHR** | **Position (Mb)** | **nSNP** | **Gene name** |
| --- | --- | --- | --- | --- |
| BER | 1 | 61.06 -63.01 | 42 | MANEA |
|  | 6 | 5.52-6.51 | 52 | CDH13/MPHOSPH6/HSD17B2/SDR42E1/PLCG2/ |
|  | 8 | 77.20-80.83 | 67 | GA7B/FAM160A1/SH3D19/PRSS48/RPS3A/LRBA/MAB21L2/DCLK2/IQCM/NR3C2/ARHGAP10 |
|  | 9 | 129.04-130.63 | 81 | PTPM14/SMYD2/PROX1/RPS6KC1/ANGEL2/VASH2/FLVCR1/TATDN3/NSL1 |
|  | 14 | 65.87-68.09 | 48 | ZNF365/ADO/EGR2/NRBF2/JMJD1C/MIR1296/REEP3 |
|  | 15 | 15.76-17.03 | 40 | DARS/MCM6/LCT/UBXN4/R3HDM1/MIR128-1/ZRANB3/RAB3GAP1/MAP3K19/CCNT2/ZRANB3 |
|  | 16 | 69.57-70.29 | 27 | GRIA1 |
|  | 17 | 4.39-5.63 | 54 | FGF20/MICU3/ZDHHC2/CNOTT/VPS37A/MTMR7/SLC7A2/PDGFRL/MTUS1/FGL1 |
| LAN | 2 | 73.88-79.33 | 61 | NFIC/GNG7/CNO76/RNF130/KDM4B |
|  | 7 | 48.16-50.08 | 54 | RASGRF1/MIR184/ANKRD34C/TMED3/KIAA1024/BCL2A1/ZFAND6/FAH/ARNT2/ABHD17C/CEMIP/MESDC2/CFAP161/IL16 |
|  | 14 | 123.35-124.06 | 27 | TCF7L2/HABP2/NRAP/CASP7 |
|  | 14 | 124.21-124.28 | 6 | NHLRC2 |
| HZ | 8 | 65.43-65.48 | 2 | UBA6/GNRHR |
|  | 8 | 66.23-67.50 | 25 | UGT2B31/SULT1E1/CSN1S1/CSN2/STATH/CSN1S2/PRR27/ODAM/CSN3/CABS1/AMTN/AMBN/ENAM/JCHAIN/UTP3/RUFY3 |
| PIE | 2 | 7.85-9.13 | 50 | RTN3/SLC3A2/BSCL2/FERMT3/OTUB1/PLA2G16/SLC22A6/STX5/FKBP2/RCOR2 |
|  | 4 | 16.94-19.44 | 96 | SHAS2/SNTB1/MTBP/MRPL13/COL14A1/DEPTOR/DSCC1/TAF2/ENPP2 |
|  | 6 | 44.69-46.60 | 38 | FXYD7/GAPDHS/LRFN3/FFAR3/TBCB/COX6B/DMKN |
|  | 8 | 65.55-68.86 | 47 | SLC4A4/RUFY3/NPFFR2/PCK/MOB1B |
|  | 12 | 2.37-2.91 | 25 | CCDC40/TBC1D16/CBX4/CBX8/CBX2/ENPP7 |
|  | 12 | 3.04-3.97 | 28 | RBFOX3/ENGASE/C1QTNF1/CANT1/LGALS3BP/TIMP2/CEP295NL/USP36/CYTH1/DNAH17/PGS1/SOCS3/TMEM235/BIRC5/AFMID/TK1/SYNGR2/TMC8/TMC6/TNRC6C |
|  | 13 | 29.59-29.89 | 10 | ALS2CL/TMIE/PRSS50/PRSS46/MYL3/PTHIR/CCDC12/NBEAL2/SETD2 |
|  | 16 | 49.85-52.75 | 60 | CPEB4/BOD1/STC2/BNIP1/CREBRF/ATP6VOE1/RPL26L1/ERGIC1/DUSP1/NEURL1B/SH3PXD2B/UBTD2/EFCAB9/STK10/FBXW11/SMIM23/FGF18 |
| WNHUAZ | 1 | 111.18-117.45 | 86 | MYO1E/LIPC/CGNL1/TCF12/RFX7/PRTG |
|  | 1 | 142.77-145.30 | 41 | CHRNA7/OTUD7A/KLF13/TRPM1/MTMR10/FAN1/MPHOSPH10/MCEE/APBA2/FAM189A1/NSMCE3/TJP1/SEH1L/TARSL2 |
|  | 2 | 15.40-16.10 | 28 | LRP4/CKAP5/ZNF408/ARHGAP1/ATG13/HARBI1/AMBRA1 |
|  | 8 | 64.83-68.48 | 52 | CENPC/UBA6/STAP1/RUFY3/SLC4A4/MOB1B |
|  | 16 | 48.62-51.50 | 47 | MRPS27/PTCD2/ZNF366/TNP01/DRD1/MSX2/CPEB4/BOD1/STC2/BNIP1/CREBRF/ATP6UOE1/RPL26L1/ERGIC1/DUSP1 |

The distance between genes and ROH regions was calculated as follows: The starting coordinate of the gene minus the starting coordinate of the ROH region; all candidate genes are located in the ROH region.

**Supplemental Table 3 Pig QTLs located in genomic regions with a high frequency of ROHs**

| **Breed** | **CHR** | **Position (Mb)** | **Traits related with QTLs** | **QTL name (QTL ID)** |
| --- | --- | --- | --- | --- |
| BER | 1 | 61.06 -63.01 | Meat&Carcass/ Production | Loin muscle area(46); Average daily gain(16885); Carcass weight(16896); Meat color(3980); muscle protein percentage(3838); Percentage type IIa fibers(7015); |
|  | 6 | 5.52-6.51 | Exterior/Meat&Carcass/Production | Vertebra number(21304); Lean meat percentage(7632); Meat color(3921); Loin muscle area(5698); Average daily gain(344); Percentage type IIb fibers(7043); |
|  | 8 | 77.20-80.83 | Production/Exterior/ Meat&Carcass | Residual feed intake(31321); Thoracolumbar vertebra number(64740); Estimated carcass lean content(5707); Body weight(21254); |
|  | 9 | 129.04-130.63 | Production/  Meat&Carcass | Body weight(151336); Carcass weight(12786);Average daily gain(2896); Carcass length(29706); Diameter of type IIa muscle fibers(7017); |
|  | 14 | 65.87-68.09 | NA | NA |
|  | 15 | 15.76-17.03 | Meat&Carcass | Meat color score(3009); Shear force(3011) |
|  | 16 | 69.57-70.29 | Production | Days to 100 kg(62351); Average daily gain(16858); body weight(3214) |
|  | 17 | 4.39-5.63 | Meat&Carcass/  Production | Intramuscular fat content(193559); Average daily gain(28911); Body weight(5263) |
| LAN | 2 | 73.88-79.33 | Exterior/Meat&Carcass/ Production | Hip structure(125570); Average backfat thickness(28083); Body width(125542); Body depth(125495); Lean meat percentage(216174); Chest width(223627); Carcass weight(9825); |
|  | 7 | 48.16-50.08 | Meat&Carcass/ Production/ Exterior | Average backfat thickness(9839); Average daily gain(3834); Carcass weight(12766); Carcass length(1066); Body weight(1076); Loin muscle area(1053); Lumbar vertebra number(21308); |
|  | 14 | 123.35-124.06 | Meat&Carcass | Diameter of type IIa/IIb muscle fibers(2827/2828); Loin muscle area(12082); |
|  | 14 | 124.21-124.28 |  | Loin muscle depth(12112); |
| HZ | 8 | 65.43-65.48 | Health/ Reproduction | Red blood cell count(22148); CD4-positive/CD8-positive leukocyte percentage(17844/17845); Teat number(4253); Corpus luteum number(492); |
|  | 8 | 66.23-67.50 | Meat&Carcass/Health/ Reproduction | Palmitoleic acid content(168374); Mycoplasmal pneumonia susceptibility(120287); Teat number(124206); Mean corpuscular volume(21515); CD4-positive/CD8-positive leukocyte percentage(17844/17845); Red blood cell count(15053); Corpus luteum number(492); |
| PIE | 2 | 7.85-9.13 | Reproduction/ Meat&Carcass/  Production | Litter size(130418); Average backfat thickness(153586); Average daily gain(5932); Feed conversion ratio(5160); Body weight(5935); Carcass weight(4245); Carcass length(4112); |
|  | 4 | 16.94-19.44 | Reproduction/ Meat&Carcass/  Production | Litter size(151324); Body length(125493); Feed conversion ratio(5162); Carcass weight(21362); Body weight(449); Average daily gain(321); |
|  | 6 | 44.69-46.60 | Meat&Carcass/  Production | Drip loss(36177); Lean meat percentage(216185); Loin muscle area(172594); Loin muscle depth(172612); Average daily gain(3937); |
|  | 8 | 65.55-68.86 | Meat&Carcass/  Production | Average daily gain(498); Loin muscle area(3264); Body weight(29699); |
|  | 12 | 2.37-2.91 | Production/Exterior | Average daily gain(171471); Thoracic vertebra number(21312); Vertebra number(21311); |
|  | 12 | 3.04-3.97 | Meat&Carcass/  Production/Exterior | Lean meat percentage(1127); Carcass length(5716); Thoracic vertebra number(21312); Vertebra number(21311); |
|  | 13 | 29.59-29.89 | Meat&Carcass/  Production | Loin muscle area(5499); Lean meat percentage(3076); Body weight(21843); Average daily gain(5721); |
|  | 16 | 49.85-52.75 | Meat&Carcass/  Production | Average backfat thickness(160475); Average daily gain(28900); body weight(3215); |
| WNHUAZ | 1 | 111.18-117.45 | Meat&Carcass/Health/ Reproduction | Palmitoleic acid content(168357); Lymphocyte number(107353); Total number born alive(140121); Marbling(78); Hemoglobin(7215); LDL cholesterol(17713); Teat number(6481); |
|  | 1 | 142.77-145.30 | Meat&Carcass/Health/ Reproduction | Intramuscular fat content(17747); Average backfat thickness(216335); Segmented neutrophil number(5480); White blood cell number(4262); Gestation length(10617); |
|  | 2 | 15.40-16.10 | Meat&Carcass/Health/ Reproduction | Cholesterol level(218043); Unsaturated fatty acid content(218044); Teat number(593); Average backfat thickness(923); |
|  | 8 | 64.83-68.48 | Meat&Carcass/Health/ Reproduction | Palmitoleic acid content(168374); Mycoplasmal pneumonia susceptibility(120287); Teat number(124208); Red blood cell count(22148); Corpus luteum number(643); Bilirubin level(6374); |
|  | 16 | 48.62-51.50 | Meat&Carcass/  Reproduction | Intramuscular fat content(147541); Average backfat thickness(160475); Teat number(8812); Polyunsaturated fatty acid to saturated fatty acid ratio(213834); |

The distance between genes and ROH regions was calculated as follows: The starting coordinate of the gene minus the starting coordinate of the ROH region;

**Supplemental Table 4 The summary of Sample information for each breed**

| **Population** | **Beed** | **N^1^** | **Birth date** | **Breeding model** | **Feeding model** | **Region** |
| --- | --- | --- | --- | --- | --- | --- |
| AHIPS | YZ | 30 | 2017-2019 | Artificial insemination/natural mating | free-ranging | Guangde, Anhui |
|  | WHHZ | 30 | 2017-2019 | Artificial insemination/natural mating | free-ranging | Jixi, Anhui |
|  | HZ | 30 | 2016-2019 | Artificial insemination/natural mating | free-ranging | Dingyuan, Anhui |
|  | WNHUAZ | 30 | 2017-2019 | Artificial insemination/natural mating | free-ranging | Huangshan, Anhui |
|  | LB | 30 | 2017-2019 | Artificial insemination/natural mating | free-ranging | Anqing, Anhui |
| WECPS | LAN | 30 | 2017-2018 | Artificial insemination | stable breeding | Chizhou, Anhui |
|  | DUR | 30 | 2017-2019 | Artificial insemination | stable breeding | Chizhou, Anhui |
|  | PIE | 30 | 2017-2018 | Artificial insemination | stable breeding | Chizhou, Anhui |
|  | LWY | 50 | 2017-2019 | Artificial insemination | stable breeding | Chizhou, Anhui |
|  | BER | 30 | 2017-2019 | Artificial insemination | stable breeding | Bozhou, Anhui |

^1^: Number of Individuals, N

**Supplementary** **Figure 1. The percentage of chromosome coverage (%) by ROHs in each breed**. YZ, Wei pigs; WNHZ, Wannan black pigs; HZ, Huai pigs; WNHUAZ, Wannanhua pigs; LB, Six White pigs; LAN, Landrace pigs; DUR, Duroc pigs; PIE, Piétrain pigs; LWY Large White pigs; BER, Berkshire pigs;

**Supplementary** **Figure 2. Manhattan plot of the occurrence (%); of SNPs in ROHs in ten pig breeds.** The x-axis represents the SNP genomic coordinate in each chromosome, and the y-axis shows the proportion of overlapping ROHs shared among individuals, based upon the number in population. Colourful data points indicate SNPs, and the dashed line represents the 40% threshold. YZ, Wei pigs; WNHZ, Wannan black pigs; HZ, Huai pigs; WNHUAZ, Wannanhua pigs; LB, Six White pigs; LAN, Landrace pigs; DUR, Duroc pigs; PIE, Piétrain pigs; LWY Large White pigs; BER, Berkshire pigs.
